# Initial Phase NT-proBNP, but Not Copeptin and High-Sensitivity Cardiac Troponin-T Yielded Diagnostic and Prognostic Information in Addition to Clinical Assessment of Out-of-Hospital Cardiac Arrest Patients With Documented Ventricular Fibrillation

**DOI:** 10.3389/fcvm.2018.00044

**Published:** 2018-06-07

**Authors:** Reidun Aarsetøy, Hildegunn Aarsetøy, Tor-Arne Hagve, Heidi Strand, Harry Staines, Dennis W. T. Nilsen

**Affiliations:** ^1^Division of Cardiology, Stavanger University Hospital, Stavanger, Norway; ^2^Department of Medicine, Stavanger University Hospital, Stavanger, Norway; ^3^Multidisciplinary Laboratory Medicine and Medical Biochemistry, Akershus University Hospital, Lørenskog, Norway; ^4^Sigma Statistical Services, Balmullo, United Kingdom; ^5^Department of Science, University of Bergen, Bergen, Norway

**Keywords:** out-of-hospital sudden cardiac arrest, diagnostics, prognosis, ultrasensitive copeptin, high-sensitive Troponin-T, NT-proBNP

## Abstract

**Aim:**

Sudden cardiac arrest (SCA) secondary to ventricular fibrillation (VF) may be due to different cardiac conditions. We investigated whether copeptin, hs-cTnT and NT-proBNP in addition to clinical assessment may help to identify the etiology of SCA and yield prognostic information.

**Methods and Results:**

EDTA-blood was collected prior to or at hospital admission from patients with SCA of assumed cardiac origin. Clinical data were obtained from hospital records. VF was the primary heart rhythm in 77 patients who initially were divided into 2 groups based on whether they had an ischemic or non-ischemic mechanism as the most likely cause of SCA. They were further divided into 4 groups according to whether or not they had a history of previous heart disease. The patients were categorized by baseline clinical information, ECG, echocardiography and coronary angiography; Group 1 (n = 43): SCA with first AMI, Group 2 (n = 10): SCA with AMI and previous MI, Group 3 (n = 3): SCA without AMI and without former heart disease, Group 4 (n = 18): SCA without AMI and with known heart disease. Copeptin and hs-cTNT did not differ between patient groups, whereas NT-proBNP was significantly higher in patients with established heart disease without AMI and differed between non-AMI and AMI. Furthermore, NT-proBNP was significantly elevated in non-survivors as compared to survivors.

**Conclusion:**

NT-proBNP provided both diagnostic and prognostic information in blood samples collected close to out-of-hospital resuscitation of VF patients, whereas copeptin and hs-cTnT failed to do so.

**Clinical Trial Registration:**

ClinicalTrials.gov, NCT02886273.

## Introduction

Sudden cardiac death (SCD) is a major cause of mortality in industrialized countries. In Europe, the incidence of out-of-hospital Emergency Medical Services (EMS) attended sudden cardiac arrest (SCA) is estimated to 81.6/100,000 person-years, 52.5% of which is presumed to have a cardiac cause (1). Prior heart disease (HD) is a major risk factor for SCA with an increase in incidence from 0.8/1,000 person-years in subjects without HD to 6.0/1,000 person-years in those with clinically recognized HD (2). Coronary artery disease (CAD) is the most common underlying HD associated with out-of-hospital cardiac arrest (OHCA), accounting for 65–70% of all SCD (3).

SCA secondary to VF may be the presenting symptom of acute myocardial ischemia or may be a consequence of scarring due to a previous myocardial infarction. Cardiac troponin (c-Tn) is the most commonly used biomarker of cardiomyocyte injury and is considered to be the gold standard for diagnosing an acute myocardial infarction (AMI) (4). Although earlier detection of AMI may be obtained by the introduction of high sensitivity (hs)-cTn assays, there still remains a troponin-blind period very early after symptom onset (5, 6) with the need for serial blood sampling to diagnose or exclude an AMI (4, 6). Furthermore, there are challenges related to the specificity of elevated levels of hs-cTn, which also may be due to other mechanisms of myocardial injury (7, 8). Therefore, measurement of hs-cTn in a single blood draw in the ambulance or at admission may not provide sufficient information for the diagnosis of AMI among patients with OHCA.

Lately, several studies have demonstrated an incremental diagnostic value of copeptin when added to conventional cTn or hs-cTn for early detection of AMI (9, 10). Copeptin is a 39-aminoacid glycosylated neuropeptide synthesized in the hypothalamus as the C-terminal part of the vasopressin prohormone and is secreted in equimolar amounts to arginine vasopressin (AVP) from the neurohypophysis. The exact function of circulating copeptin is unknown. Circulating copeptin levels have been shown to be significantly elevated during the initial phase of an AMI (5, 6, 9), most likely as a response to endogenous stress and hemodynamic changes due to myocardial ischemia (6).

Copeptin levels peak early (0–1 h) after AMI symptom onset and are already increased at the time of first medical contact in the ambulance for patients with both ST-elevation myocardial infarction (STEMI) and non-ST-elevation myocardial infarction (NSTEMI), after which this biomarker will decrease rapidly within 6–12 h (5). Copeptin used in combination with hs-cTn may therefore improve the diagnosis of AMI in very early presenters. Whether this may help to differentiate the underlying cause of SCA, is not known.

Cardiomyopathies are the second largest cause of SCD (11), and 30–50% of heart failure deaths present as SCD. N-terminal pro-B-type natriuretic peptide (NT-proBNP) is a marker of heart failure. It is secreted by cardiomyocytes in response to increased myocardial wall stress due to volume or pressure overload. It has a high negative predictive value in both the acute and non-acute settings (12).

Identification of factors that precipitate a fatal arrhythmia represent a major challenge. In this study, we hypothesized that the addition of copeptin, hs-cTnT and NT-proBNP would add valuable information with respect to etiology in OHCA-patients.

It is well known that chest pain patients with elevated troponins have a worsened prognosis (13). Copeptin has also been demonstrated to be a prognostic marker in AMI-patients (14, 15), whereas the prognostic value of troponins and copeptin in patients experiencing a cardiac arrest (CA) is less known (7, 16). B-type natriuretic peptide (BNP) has previously been shown to be an independent predictor of long-term mortality after CA (17, 18), as well as survival to hospital discharge after OHCA of cardiac origin (19). There is also a well-documented association between BNP and the short- and long-term risk of death in patients with acute coronary syndrome (ACS) (20). In our study, the objective was to evaluate the prognostic utility of hs-cTnT, copeptin and NT-proBNP in clinically categorized SCA patients. Prehospital blood sampling enabled us to include non-admitted patients without return of spontaneous circulation (ROSC), a patient category which is missed out in previous hospital-based studies.

## Methods

### Study Subjects and Design

From February 2007 until November 2010 we collected EDTA-blood from patients >18 years of age with OHCA of assumed cardiac origin, in collaboration with EMS paramedics in the Stavanger region of Norway. All OHCA patients recruited in this study received EMS provided advanced life support according to the 2005 European Resuscitation Council (ERC) guidelines with Norwegian alterations (21). 20 ml of EDTA-blood was drawn from a venous cannula during- or immediately after termination of CPR. Patients with permanent return of spontaneous circulation (ROSC) were transported to one hospital, Stavanger University Hospital, for further treatment. Patients with ROSC without a prehospital blood sample, were sampled at hospital admission. Informed consent was collected retrospectively. All survivors gave written, informed consent before leaving the hospital. If the patient did not regain consciousness before death, the next-of- kin were asked for consent on the patient’s behalf.

OHCA patients with documented VF as the primary heart rhythm were initially divided into 2 groups according to ischemic or non-ischemic mechanisms for SCA. They were further divided into 4 groups based on whether or not they had a history of heart disease. Group 1: SCA with first AMI, Group 2: SCA with AMI and previous MI, Group 3: SCA without AMI and without former heart disease and Group 4: SCA without AMI and with former heart disease. For patients with ROSC the presence or absence of an AMI was determined by applying previous and current clinical information from hospital records and from the Utstein database (22), by ST-segment analysis of the ECG according to the definition of STEMI (23) or NSTEMI (4), by echocardiography demonstrating findings of hypo- or akinetic regions of the left ventricle, and/or by identification of a culprit lesion by coronary angiography. A 12-lead ECG was recorded in all patients with ROSC, either during transport to hospital or/and at admission. Serial sampling of cTnT during the hospital stay was used to support the diagnosis of AMI. The diagnosis of AMI in non-ROSC patients was based on information of recent coronary-suspected chest pain, obtained from next-of-kin or EMS paramedics, and settled by consensus among the investigators prior to biomarker analyses.

STEMI patients were immediately transported to the catheterization laboratory for coronary angiography, and percutaneous coronary intervention (PCI) was performed according to current guidelines whenever a culprit lesion was identified. Significant coronary artery disease was defined by the presence of diameter stenosis >50%. The extent of coronary artery disease was evaluated by experienced operators during the procedure and later re-evaluated, if in doubt. Comatose patients received hypothermic treatment according to current practice.

Echocardiography was performed as soon as possible after admission, and repeated in survivors during hospitalization, acquiring data related to left ventricular function and valvular disorders.

The present study was approved by the Regional Board of Research Ethics and the Norwegian Health Authorities and conducted in accordance with the Helsinki Declaration of 1975, as revised in 1983.

## Laboratory Methods

After collection, all blood samples were centrifuged within 24 h at room- temperature or within 48 h of storage in a refrigerator. EDTA-plasma was extracted and stored in aliquots at −70 ^○^C until the analyses could be performed. Copeptin, ultrasensitive copeptin, hs-cTnT and NT-proBNP were all measured by standardized methods at the Department of Multidiciplinary Laboratory Medicine and Medical Biochemistry, Akershus University Hospital.

Copeptin and ultrasensitive copeptin were measured with Time-Resolved Amplified Cryptate Emission (TRACE)-technology using a Kryptor Compact Plus Instrument (B.R.A.H.M.S GmbH, Thermo Fisher Scientific, Hennigsdorf, Germany). For copeptin the assay has a detection limit of 4.8 pmol/L and a functional assay sensitivity of <12 pmol/L, assessed as interassay precision of 20% CV. Ultrasensitive copeptin has a limit of detection of 0.9 pmol/l and a functional assay sensitivity of <2.0 pmol/L with interassay precision of 20% CV. The cut-off recommended for ruling out AMI at admission is 14 pmol/L for copeptin and 10 pmol/L for ultrasensitive copeptin.

Hs-cTnT (Roche Diagnostics) was measured with electrochemiluminescence immunoassay (ECLIA) on a Cobas e602 device. This assay has a limit of detection of 3 ng/L with a measuring range of 3–10,000 ng/L. Reference limit based on the 99th percentile was >14 ng/L [99th percentile and 10% coefficient of variation (CV)].

NT-proBNP was measured by a “sandwich” ECLIA method on a Cobas e602 device (Roche Diagnostics). A value <35 pmol/L precludes heart failure.

Analyses of creatinine, electrolytes, hs-CRP and lipids were performed along with the hospital`s routine samples. Laboratory personnel performing the analyses were all blinded with respect to categorization of patients.

## Statistics

Baseline characteristics for the different groups of SCA-patients are presented as median with interquartile range (25–75th percentile) due to a relative small number of patients in each group. Values for copeptin, ultrasensitive copeptin, hs-cTnT and NT-proBNP are given with both median (with interquartile range) and mean ± SD. The latter was used for comparing Group 1 + 2 with Group 4, applying a one-way ANOVA after log transformation. Group 3 was excluded from analysis due to small group size. We also used ANOVA after log transformation to compare Groups 1, 2 and 4. For the total population, as well as for Group 1 + 2 and Group 4, respectively, the same parameters were compared between survivors and non-survivors using the Mann-Whitney U Test. Non-survivors were defined as death prior to admission or during the hospital stay.

Patients were divided into quartiles according to the NT-proBNP values. A Cox regression model containing NT-proBNP was fitted for the analysis of death within 30 days. Hazard ratios with 95% confidence intervals were calculated for each of the higher quartiles against quartile 1. The Kaplan-Meier product limits were used for plotting the times to event and the Log Rank test was used to test for the equality for the survival curves.

Spearman’s correlation coefficient was calculated to identify potential pair-wise relations between the different variables. The statistical analyses were performed with the statistical package SPSS version 24.0. All tests were 2-sided with a significance level of 5% without multiplicity adjustment.

## Results

**Figure 1:**
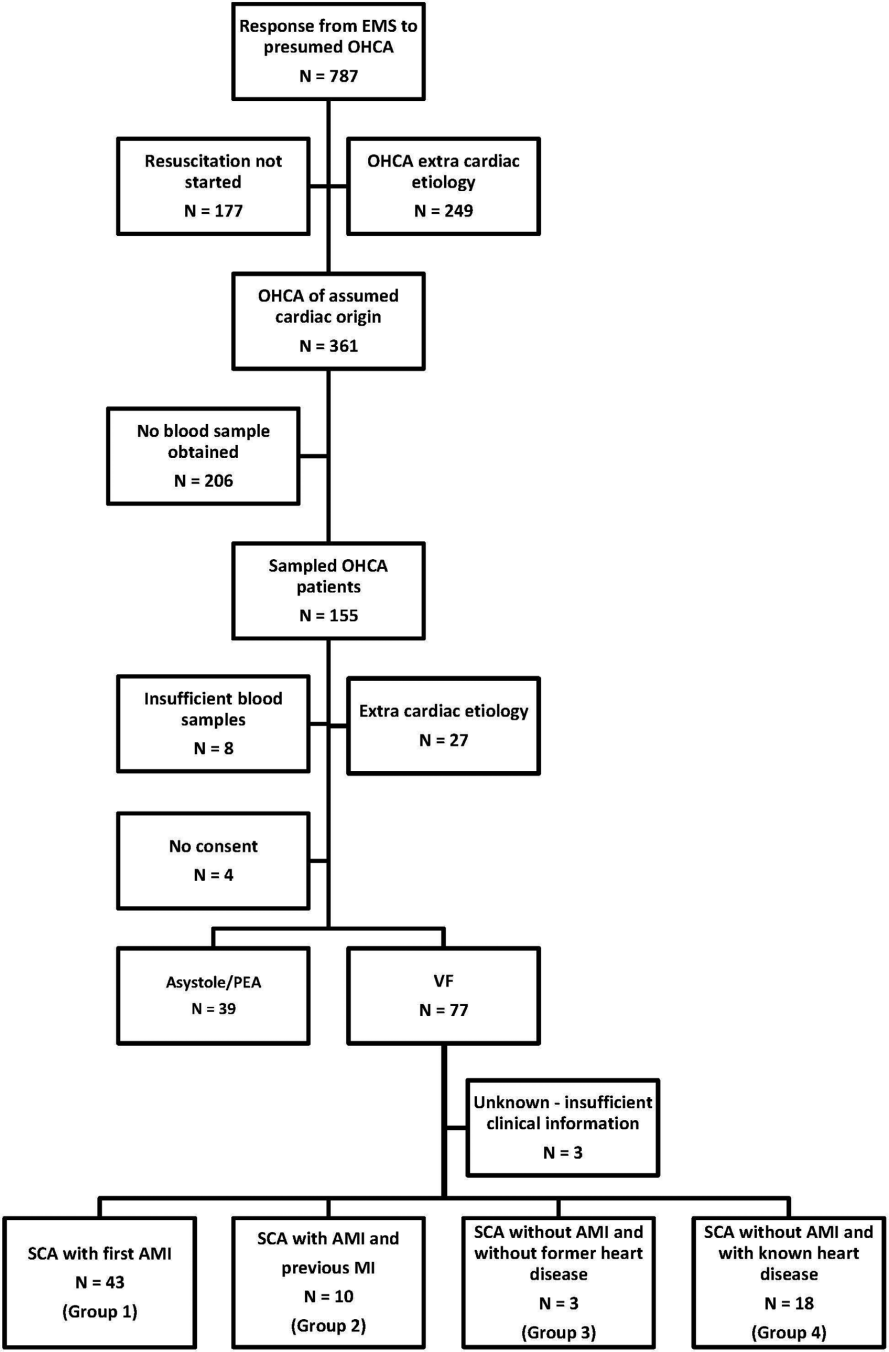
Flow-chart displaying selection and classification of patients with OHCA-VF recruited between February 2007 and November 2010.

During the study period, the EMS responded to 787 emergency calls to patients with presumed OHCA. 361 patients suffered cardiac arrest (CA) of assumed cardiac origin, defined according to the Utstein definitions (14) and were eligible for inclusion in this study (24). We managed to obtain blood samples from 155 of these patients. Retrospectively, a total of 39 patients had to be excluded for different reasons as given in Figure 1. The final population of 116 patients with OHCA of cardiac origin was further divided into two groups according to the primary heart rhythm recorded by the EMS; 39 patients with asystole or pulseless electrical activity (PEA) and 77 patients with VF (Figure 1).

The group of VF-patients was classified according to the presence of an AMI. Out of the remaining 74 patients, 53 patients had signs of AMI while 21 patients suffered SCA without signs of AMI. Baseline characteristics of patients with ischemic versus non-ischemic mechanisms for VF are presented in Table 1. The patients were further divided into 4 groups based on whether or not they had previously known heart disease. 43 patients had SCA as a manifestation of their first AMI (Group 1) and 10 patients suffered SCA as a consequence of a recurrent AMI (Group 2). Among the 21 patients with non-ischemic CA there were three subjects who had no clinically suspected former heart disease (Group 3). The latter group consisted of two males and one female, age ranging from 18 to 54 years, all with preserved EF and one presenting with WPW syndrome, and all surviving to hospital discharge. The number of patients in Group 3 was too low for further statistical evaluation. Eighteen of the non-ischemic CA-patients had evidence of prior heart disease, including coronary artery disease (CAD) and/or congestive heart failure (CHF) (Group 4) (Figure 1). A separate baseline table for individual groups, excluding Group 3, is presented in Table S1.

**Table 1:** Baseline characteristics of patients suffering out-of-hospital cardiac arrest associated with documented ventricular fibrillation.

** **	**SCA with AMIGroup 1 and 2** **(n = 53)**	**SCA without AMIGroup 3 and 4** **(n = 21)**	***P*-value **
Men	47 (89%)	19 (90%)	0.320 [1]
Age (median, years)	60 (49–69)^1^	70 (63–81)^1^	0.011 [2]
BMI (mean, kg/m^2^)	27.6^2^	27.6^2^	0.874 [3]
**Symptoms prior to SCA**			**< **0.001 [1]
Chest pain	29 (55%)	1 (5%)	
Dyspnoea	1 (2%)	2 (9,5%)	
Palpitations/syncope	0	1 (5%)	
Asymptomatic	8 (15%)	7 (33%)	
Unknown	15 (28%)	10 (48%)	
**ECG findings **			N/A
STEMI	35 (66%)	0	
NSTEMI	10 (19%)	0	
Unknown	8 (15%)	0	
**Ejection fraction **(median, %)	42.5 (30–60)^1,4^	45 (25–60)^1,5^	0.582 [2]
**Coronary angiography**			0.003 [1]
Normal	0^6^	4 (29%)^7^	
1-vessel disease	20 (44%)^6^	3 (21%)^7^	
2-vessel disease	11 (24%)^6^	1 (7%)^7^	
3-vessel disease	14 (31%)^6^	6 (43 %)^7^	
**Coronary intervention**			
LAD	26 (58%)^6^	1 (7%)^7^	< 0.001 [1]
CX	11 (24%)^6^	0	0.051 [1]
RCA	7 (16%)^6^** **	1 (7%)^7^	0.666 [1]
**Hypothermic treatment**	34 (69%)^8^	15 (83%)^9^	0.356 [1]
**Implantation of ICD**	1 (2%)	10 (48%)	< 0.0001 [1]
**Death prior to discharge**	21 (40%)	9 (43%)	0.799 [1]
**Previous history**			
Angina pectoris	5 (11%)^10^	3 (16%)^11^	0.683 [1]
Myocardial infarction	10 (19%)^12^	11 (52%)	0.009 [1]
Heart failure	1 (2%)^13^	15 (71%)	< 0.001 [1]
Previous CABG	2 (4%)^12^	4 (19%)	0.053 [1]
Previous PCI	5 (10%)^12^	3 (14%)	0.682 [1]
Hypertension	19 (39%)^8^	12 (60%)^14^	0.120 [1]
Mitral insufficiency	3 (6%)^15^	13 (62%)	< 0.001 [1]
Diabetes mellitus	6 (13%)^16^	3 (14%)	1,0 [1]
Hypercholesterolemia	27 (54%)^17^	5 (26%)^11^	0.058 [1]
Smoking			
Current smoking	11 (30%)^18^	6 (35%)^19^	
Ex-smoker	21 (57%)^18^	6 (35%)^19^	
Family history	21 (66%)^20^	6 (46%)^7^	0.317 [1]
**Medication prior to admission**			
Beta-blocker	4 (10%)^21^	10(48%)	0.003 [1]
Ca-blocker	8 (20%)^21^	6 (29%)	0.524 [1]
ACEI/ARB	6 (15%)^21^	15 (71%)	< 0.001 [1]
Diuretics	3 (7%)^21^	13 (62%)	< 0.001 [1]
ASA	8 (20%)^21^	7 (33%)	0.347 [1]
Warfarin	1 (2%)^21^	9 (43%)	0.0001 [1]
Statins	11 (26%)^22^	13 (62%)	0.0121 [1]
Anti-arrhythmics	0	0	N/A
**Baseline blood samples (median)**			
Potassium (mmol/L)	3.9(3.4–4.1)^1,15^	4.2(3.4–4.5)^1,19^	0.214 [2]
Creatinine (umol/L)	98 (86–114)^1^	121 (89–139)^1^	0.035 [2]
Total-cholesterol (mmol/L)	5.0 (3.9–6.1)^1^	3.9 (3.2–4.2)^1^	< 0.001 [2]
Glucose (mmol/L)	14.2 (10.3–19.4)^1,21^	13.5 (8.8–14.7)^1,11^	0.240 [2]
hs-CRP (mg/L)	1.9 (1.1–3.7)^1^	2.0 (1.1–10.8)^1 ^	0.387 [2]
hs-cTNT (ng/L)	97.4 (25.8–272)^1 ^	51.6 (26.1–125)^1^	0.185 [2]
Copeptin (pmol/L)	558 (261–1,029)^1,17^	454 (175–517)^1,11^	0.088 [2]
Copeptin ultrasensitive (pmol/L)	464 (241–833)^1^	389 (133–500)^1,14^	0.105 [2]
NT-proBNP (pmol/L)	28.2 (13.8–76.8)^1^	165 (59.4–340)^1^	0.000 [2]

Categorical data are given as n (%). Median values of continuous datagiven with 25th and 75th percentiles in parentheses(interquartile range). Nd = no data. ^1^Median with range, ^2^n = 29, ^3^n = 14, ^4^n = 30, ^5^n = 11, ^6^n = 45, ^7^n = 13, ^8^n = 49, ^9^n = 18, ^10^n = 46, ^11^n = 19, ^12^n = 52, ^13^n = 51, ^14^n = 20, ^15^n = 47, ^16^n = 48, ^17^n = 50, ^18^n = 37, ^19^n = 17, ^20^n = 32, ^21^n = 41, ^22^n = 42. [1] Fisher’s exact test, [2] Kruskal-Wallis test, [3] One-way analysis of variance.

ACEI, angiotensin converting enzyme inhibitor; ARB, angiotensin II receptor blocker; ASA, acetylsalisylic acid; BMI, body mass index; CABG, coronary artery bypass grafting; CAD, coronary artery disease; CX, circumflex; ECG, electrocardiography; HDL, high density lipoprotein; HF, heart failure; hsCRP, high-sensitivity C-reactive protein; ICD, implantable cardioverter defibrillator; LAD, left anterior descending artery; RCA, right coronary artery; MI, myocardial infarction; NSTEMI, non-ST-elevation myocardial infarction; NT-proBNP, N-terminal-pro brain natriuretic peptide; PCI, percutaneous coronary intervention; SCD, sudden cardiac death; SD, standard deviation; STEMI, ST-elevation myocardial infarction; hsTnT, high-sensitivity troponin-T.

There was a male dominance in all groups. Median age ranged from 50 to 74 years, with the oldest patients belonging to Group 2 and 4. Chest pain was the most frequent symptom prior to SCA in patients diagnosed with AMI, and 66% of AMI was classified as STEMI. All survivors with AMI had findings of CAD by coronary angiography. 1-vessel disease was the most common finding among patients with first time AMI, while 3-vessel disease was more common in patients with recurrent MI. Left anterior descending coronary artery (LAD) was the most frequent infarct-related artery. Patients with known heart disease (Group 2 and 4) had a lower ejection fraction (EF) estimated by echocardiography, a higher rate of mitral insufficiency and were taking more medications than the previously healthy VF-patients. In SCA patients with established heart disease without AMI (Group 4), 83% had a former diagnosis of heart failure and 61% had previous MI.

Blood samples were harvested in 42 patients during resuscitation and in 35 patients at hospital admission, with a median time from CA to blood sampling of 40 and 74 min, respectively. The highest level of hs-cTnT (246.0 ng/L, SD 450.7) was detected in SCA-patients with an AMI (Group 1 and 2). There was also a substantial release of hs-cTnT (102.1 ng/L, SD 115.1) in non-AMI patients in Group 4, resulting in no statistically significant inter-group difference (Table 2). Likewise, we also found high values of conventional and ultrasensitive copeptin in both AMI and non-AMI patients in our study, with no statistically significant difference (Table 2). The only biomarker demonstrating a highly significant difference in this study population was NT-proBNP; Group 4 having a significantly higher mean value of 405.1 pmol/L (SD 569.9) as compared to the other two groups (*p* = 0.001) (Table 3). Corresponding values reported as median and interquartile range, are given in Table 1.

**Table 2:** Plasma concentrations of biomarkers for out-of-hospital ventricular fibrillation in acute myocardial infarction (Group 1 + 2) as compared to patients with established cardiac disease without acute myocardial infarction (Group 4).

	** SCA with AMI Group 1 and 2 Mean (SD)**	**SCA without AMI Group 4 Mean (SD)**	***P*-value**
**Conventional copeptin (pmol/L)**	719.4^* ^(587.0)	438.1^*†* ^(302.9)	0.111
**Ultrasensitive copeptin (pmol/L)**	635.8 (542.1)	363.4^*‡* ^(240.8)	0.097
**hs-cTnT (ng/L)**	246.0 (450.7)	102.1 (115.1)	0.332
**NT-proBNP (pmol/L)**	67.06 (114.4)	405.1 (569.9)	0.000

Number of patients available for analyses: *n = 50, ^†^*n* = 16, ^‡^*n* = 17.

**Table 3:** Plasma concentrations of biomarkers in patients with out-of-hospital ventricular fibrillation following an acute myocardial infarction (MI), separating those with first-time (Group 1) and repeated MI (Group 2) as compared to patients with established cardiac disease without acute myocardial infarction (Group 4).

	**Group 1 Mean (SD)**	**Group 2 Mean (SD)**	**Group 4 Mean (SD)**	***P*-value**
**Conventional copeptin (pmol/L)**	666.7^* ^(579.6)	996.1^*† *^(583.4)	438.1^*‡ *^(302.9)	0.089
**Ultrasensitive copeptin (pmol/L)**	587.2 (536.5)	844.8 (542.8)	363.4^*§ *^(240.8)	0.085
**hs-cTnT (ng/L)**	274.5 (490.1)	123.5 (179.4)	102.1 (115.1)	0.333
**NT-proBNP (pmol/L)**	68.24 (123.6)	61.99 (66.07)	405.1 (569.9)	0.001

Number of patients available for analyses: *n = 42, ^†^*n* = 8, ^‡^*n* = 16, ^§^*n* = 17.

**Figure 2:**
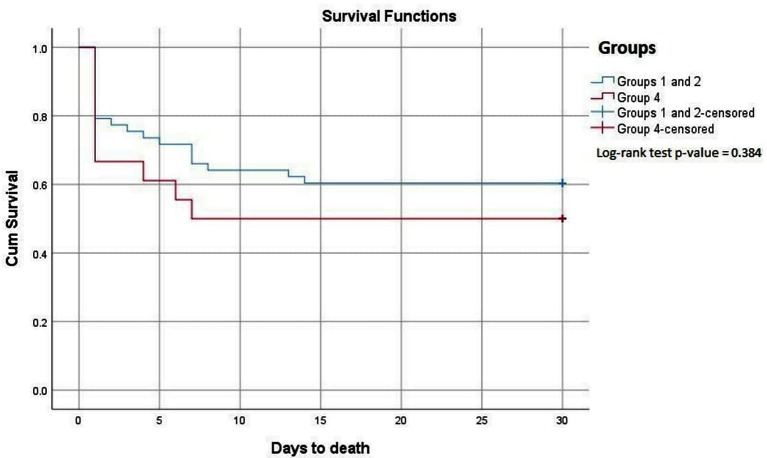
Kaplan-Meier plots for the cumulative risk for total mortality in OHCA patients, comparing Groups 1 and 2 combined with Group 4.

Prior to or early after hospital admission, 33 out of 77 patients (43%) had died, 40% in the ischemic group (Groups 1 + 2) and 43% in the non-ischemic group (Group 4). All 44 patients surviving until discharge were alive 30 days following hospitalization. Kaplan-Meier survival curves for Groups 1 + 2 as compared to Group 4 are shown in Figure 2. The survival curve for Groups 1 + 2 was not statistically different from that for Group 4 (Log-rank test; *p* = 0.384). Patients suffering from their first MI had the lowest mortality rate with 65% surviving to discharge. For patients with known heart disease (Group 2 and 4) the mortality rates were 60 and 50%, respectively. Survival curves for each separate group are shown in Supplementary data, Figure S1. Among the given clinical risk factors (Table 1), the history of diabetes was found to be associated with a high mortality rate, as 7 out of 9 of these patients died.

**Figure 3:**
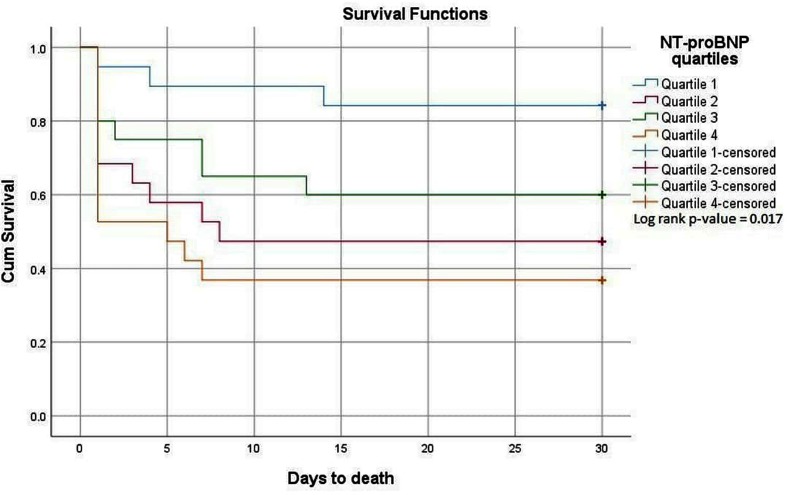
Kaplan-Meier plots for the cumulative risk for total mortality in OHCA patients according to NT-proBNP quartiles.

In a subgroup analysis, we compared the levels of copeptin, hs-cTnT and NT-proBNP, respectively, in survivors and non-survivors in the total population, as well as for patients with (Group 1 and 2) and without an AMI (Group 4). Levels of ultrasensitive copeptin, conventional copeptin and hs-cTnT did not differ between the two outcomes. Survival curves for conventional copeptin (log-rank test *p*-value = 0.398) and hs-cTNT (log-rank test *p*-value = 0.287) are presented as Supplementary Figures S2, S3, respectively. In the total population, however, NT-proBNP was significantly elevated in non-survivors, with a median value of 86.3 (27.2–189.3) pmol/L as compared to 29.9 (11.3–99.0) pmol/L in survivors (*p* = 0.024) (Table 4). Survival curves for NT-proBNP quartiles in the total population are shown in Figure 3. The hazard ratio for quartile 4 compared to quartile 1 was 5.37 (95% CI 1.51–19.16); *P* = 0.010. The HR for quartile 2 was 4.08 (CI 1.12–14.9); *p* = 0.033, and for quartile 3 the HR was 2,83 (CI 0.75–10.7); *p* = 0.125. Within-group analysis demonstrated a statistically significant difference in NT-proBNP with respect to survival when combining Group 1 and 2 (*p* = 0.038), whereas no within-group difference was found in Group 4 (Table 4).

**Table 4:** Concentrations of biomarkers for survivors and non-survivors in thedifferent patient groups.

** **	** **	Conventional copeptin^* ^(pmol/L)	**Ultrasensitive copeptin^† ^(pmol/L)**	** hs-cTnT(ng/L)**	** NT-proBNP(pmol/L)**
**Total**	**Survivors(n = 44)**	454.0 (218.9–1029.0)	388.0 (194.9–825.0)	100.3 (21.0–296.8)	29.9 (11.3–99.0)
	**Non-survivors(n = 30)**	517,2 (358.5–901.6)	463.1 (274.0–739.3)	72.0 (26.0–212.6)	86.3 (27.2–189.3)
	***P*-value**	0.240	0.420	0.360	0.024
**Group 1 and 2**	**Survivors (n = 32)**	453.7 (243.8–919.9)	377.7 (207.4–830.9)	125.3 (36.7–320.9)	25.4 (10.4–52.5)
	**Non-survivors (n = 21)**	709.3 (488.5–1,222)	702.5 (397.2–1,015)	71.0 (16.7–218.7)	61.5 (19.9–115.2)
	***P*-value**	0.101	0.151	0.114	0.038
**Group 4**	**Survivors(n = 9)**	454.0 (161.9–1,045.0)	422.8 (91.6–700.8)	51.6 (17.8–133.1)	163.7 (101.3–339.5)
	**Non-survivors(n = 9)**	358.9 (263.7–513.4)	286.0 (170.5–477.9)	73.0 (27.8–220.7)	217.6 (108.3–983.0)
	***p*-value**	1.00	0.740	0.300	0.610

Number of patients available for analyses: ^*^Total: n = 69, Group 1 and 2: n = 50, Group 4: n = 16, ^†^Total: n = 73, Group 1 and 2: n = 53, Group 4: n = 17.

**Figure 4:**
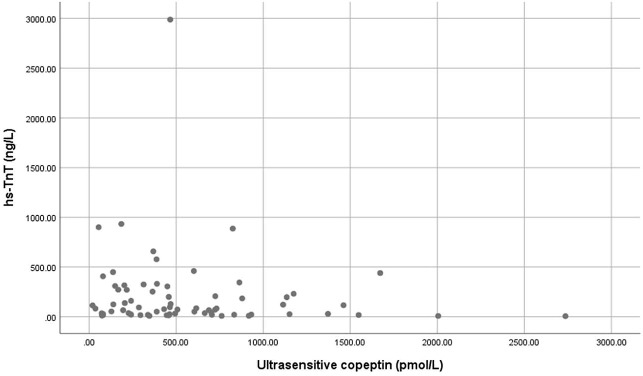
Scatter plots for hs-cTnT versus Ultrasensitive copeptin.

There was no rank correlation between ultrasensitive or conventional copeptin and hs-cTnT, neither for hs-cTnT and NT-proBNP. Furthermore, we performed scatter plots for hs-cTnT versus ultrasensitive copeptin (Figure 4), remaining scatter plots are displayed in Supplementary data Figure S4, S5.

## Discussion

During our study period, the EMS responded to 787 cases of presumed OHCA in the Stavanger-region and a resuscitation attempt was started in 610 of these (24), yielding an annual incidence of 52 EMS-assisted OHCA cases per 100,000 inhabitants, out of which 59% were assumed to be of cardiac origin. Thus, the population from which our final study participants were recruited seems to be highly representative of the general Norwegian OHCA-population (25). The higher proportion of VF (66.4%) in our study as compared to the 26% reported for the total Stavanger population by the Norwegian Cardiac Arrest Registry (25) is likely due to more frequent blood sampling among ROSC as compared to non-ROSC patients. This might also explain the high percentage of survival (57%) among our OHCA-VF patients as compared to the 30 days’ survival rate of 44% for the total Norwegian population (25) which is even higher than the survival rate of approximately 23% in the general European population (1).

We recorded high levels of both conventional- and ultrasensitive copeptin in both ischemic and non-ischemic groups, probably due to the fact that copeptin is a marker for AVP release, a stress-hormone which is shown to increase markedly in patients resuscitated from SCA (26). SCA is a severe stress condition with circulatory collapse and global hypoxia, by which the hypothalamic-pituitary-adrenal axis is activated through various mechanisms, explaining the general increase of copeptin in our VF-patients and the lack of discriminatory utility with respect to AMI.

Hs-cTnT was found to be elevated in both ischemic and non-ischemic patient groups, with a statistically non-significant trend toward a higher mean value in AMI-patients. In the FINNRESUSCI study, Røsjø et al. 2014 (7) found that admission levels of hs-cTnT were higher than the 99-percentile of the general population (14 ng/L) in all OHCA-VF/VT patients. As only 26% of patients in the two highest quartiles of hs-cTnT had a coronary angiography performed and only 17% underwent percutaneous coronary intervention (PCI), that population most likely consisted of patients with various mechanisms underlying the OHCA event, supporting the hypothesis of CPR leading to elevated levels of cardiac markers, irrespective of underlying cause.

In our study, we obtained only one blood sample in conjunction with CPR, with a median time from symptom onset to blood sampling of only 1,35 h, and we may have missed the peak values of hs-cTnT in patients with AMI. However, Oh SH et al. 2012 (8) demonstrated in a small OHCA study that the admission value of cTnI in SCA-patients with AMI did not differ as compared to SCA-patients without AMI, lending support to our results.

Neither ultrasensitive or conventional copeptin, nor hs-cTnT were found to differ significantly between survivors and non-survivors in our study (Table 4). In the FINNRESUSCI study by Røsjø et al. 2014 (7), admission hs-cTnT levels did not differ statistically between hospital non-survivors and survivors, and did not yield independent prognostic information at 1 year follow up in OHCA-VT/VF patients. In the main FINNRESUSCI study population, admission levels of copeptin were higher in patients who died in the intensive care unit (ICU), but were not predictive of 12 months survival. (16). These findings differ from those in the setting of AMI, in which copeptin has been found to be an independent predictor of adverse events following both STEMI (14) and NSTEMI (15).

 We harvested blood samples very early in the course of SCA, during which elevated levels of copeptin most likely will reflect the stress response associated with CA, rather than hemodynamic instability signaling a poorer prognosis after ROSC, such as in samples harvested up to 6 h after hospital admission in the FINNRESUSCI population (16).

In our study, VF-patients with established heart disease without AMI had a significantly higher level of NT-proBNP as compared to AMI subjects (ischemic group). In the former group, 83% of patients had a previous diagnosis of heart failure, a well-known risk factor for ventricular arrhythmias and SCD. Both BNP and NT-proBNP have been demonstrated to be useful diagnostic tools to rule out both chronic and acute heart failure (12). Previous studies have also shown that AMI alone causes an increase in circulating BNP levels (27), and Sabatine et al. 2004 (28) found an immediate rise in circulating BNP levels associated with transient myocardial ischemia during exercise stress testing. Elevation of BNP levels during myocardial ischemia may reflect increased regional ventricular wall stretch due to impaired myocardial relaxation and contractility. However, little is known about the behavior and prognostic utility of natriuretic peptides during a SCA event.

In a FINNRESUSCI substudy (17), serial measurement from baseline (<6 h following OHCA-VT/VF) up to 96 h after admission showed an increase in NT-proBNP. Patients with prolonged time to ROSC and high values of hs-TnT on admission had the highest level of NT-proBNP after 24 h, reflecting outcome in that study. In that setting, increased NT-proBNP values may be related to post resuscitation cardiogenic shock, rather than being a consequence of the CPR itself. This assumption is supported by the findings from a Japanese study, in which BNP was significantly lower in non-cardiac as compared to cardiac OHCA (29).

NT-proBNP in our study was measured in blood samples drawn very early in the course of CA, most likely reflecting the underlying heart disease, rather than CPR *per se*. This assumption is supported by the low levels of NT-proBNP in SCA patients without former heart disease (28.2 pmol/L in Group 1) which are well below the rule-out level of 35 pmol/L for acute heart failure (12). The low levels in AMI patients without previous heart disease (Group 1) indicates negligible influence of ischemia on NT-proBNP during OHCA.

Furthermore, we demonstrated a significantly higher NT-proBNP among non-survivors as compared to survivors, both in the total study population and in AMI patients, which is in accordance with BNP’s utility as an independent predictor of long-term mortality (17, 18), and of survival to hospital discharge after OHCA with VF/VT as the initial rhythm (19). Admission levels of NT-proBNP were also significantly higher in non-survivors as compared to survivors in a study by Smit et al. 2015 (30), although not predictive of 28 day-mortality, but that study included all-comers with a cardiac arrest, and patients with a non-shockable rhythm might have cancelled the prognostic utility of NT-proBNP.

In our study, NT-proBNP also served as an independent predictor of mortality in patients with OHCA and AMI, as previously shown for BNP and NT-pro BNP in relation to short- and long-term risk of death in patients with ACS (20), and in samples collected as early as 6 h from symptom onset in patients with AMI (31). Our findings are supported by those of Galvani et.al. 2004 (32) who measured NT-proBNP in samples harvested median 3 h after symptom onset in ACS.

## Strengths

The main strength of our study is the sampling of blood at a very early stage after the occurrence of OHCA and includes non-admitted patients without ROSC, a patient category which is missed out in previous hospital-based studies. Furthermore, the initial cardiac rhythm was recorded in all our patients, enabling us to select VF-patients for analysis.

## Limitations

Unfortunately, there was a selection bias due to unbalanced blood sampling in the ROSC and non-ROSC group of patients, and our study is limited to short-term observation of outcome. Recruitment was restricted to the largest ambulance centers in the area located closest to the hospital and to the medical support helicopter. It also required enough trained study personnel and timely delivery of blood samples within 24 h. Furthermore, samples were not obtained after death was declared by the “on-scene” physician.

As death during the first 30 days in patients surviving OHCA usually is related to complications, traditional multivariable analysis was not performed.

## Conclusion

Copeptin and hs-cTnT collected during or immediately after resuscitation were not found to be useful for the diagnosis of AMI or for prediction of survival in the OHCA-VF setting, whereas NT-proBNP provided both diagnostic and prognostic information.

## Ethics Statement

This study was carried out in accordance with the recommendations of the Regional Board of Research Ethics with written informed consent from all subjects. All subjects gave written informed consent in accordance with the Declaration of Helsinski. The protocol was approved by the Regional Board of Research Ethics and the Norwegian Health Authorities.

## Author Contributions

RA: Recruitment of patients, collection and preparation of blood samples, statistical analysis and writing the manuscript. HA: Design of the study, recruitment of patients, and collection and preparation of blood samples. T-AH: Analysis of biomarkers. HeS: Analysis of biomarkers. HaS: Statistical work. DN: design of the study. All authors contributed intellectually to the manuscript. 

## Conflict of Interest Statement

HaS was employed by company Sigma Statistical Services. The remaining authors declare that the research was conducted in the absence of any commercial or financial relationships that could be construed as a potential conflict of interest.

## Supplementary material

Figure S1 Kaplan-Meier plots for the cumulative risk for total mortality in OHCA patients, according to Group 1, 2 and 4.

Figure S2 Kaplan-Meier plots for the cumulative risk for total mortality in OHCA patients according to copeptin quartiles.

Figure S3 Kaplan-Meier plots for the cumulative risk for total mortality in OHCA patients according to hs-cTnT quartiles.

Figure S4 Scatter plots for hs-cTnT versus conventional copeptin.

Figure S5 Scatter plots for NT-proBNP versus conventional copeptin.

## References

[1] Berdowski J Berg RA Tijssen JG Koster RW Global incidences of out-of-hospital cardiac arrest and survival rates: systematic review of 67 prospective studies. Resuscitation (2010) 81(11):1479–87. 10.1016/j.resuscitation.2010.08.006 20828914

[2] Mozaffarian D Benjamin EJ As G Arnett DK Blaha MJ Cushman M American Heart Assosciation Statistics Committee and Stroke Statistics Subcommittee. Heart disease and stroke statistics-2015 update: a report from the American Heart Association. Circulation (2015) 131(4):e29–322. 25520374 10.1161/CIR.0000000000000152

[3] Zheng ZJ Croft JB Giles WH Mensah GA Sudden cardiac death in the United States, 1989 to 1998. Circulation (2001) 104(18):2158–63. 10.1161/hc4301.098254 11684624

[4] Roffi M Patrono C Collet J-P Mueller C Valgimigli M Andreotti F 2015 ESC Guidelines for the management of acute coronary syndromes in patients presenting without persistent ST-segment elevation. Eur Heart J (2016) 37(3):267–315. 10.1093/eurheartj/ehv320 26320111

[5] Slagman A Searle J Müller C Möckel M Temporal release pattern of copeptin and troponin T in patients with suspected acute coronary syndrome and spontaneous acute myocardial infarction. Clin Chem (2015) 61(10):1273–82. 10.1373/clinchem.2015.240580 26341999

[6] Gu YL Voors AA Zijlstra F Hillege HL Struck J Masson S Comparison of the temporal release pattern of copeptin with conventional biomarkers in acute myocardial infarction. Clin Res Cardiol (2011) 100(12):1069–76. 10.1007/s00392-011-0343-y 21766239 PMC3222827

[7] Røsjø H Vaahersalo J Hagve TA Pettilä V Kurola J Omland T Prognostic value of high-sensitivity troponin T levels in patients with ventricular arrhythmias and out-of-hospital cardiac arrest: data from the prospective FINNRESUSCI study. Crit Care (2014) 18(6):605 10.1186/s13054-014-0605-y 25490117 PMC4256726

[8] Oh SH Kim YM Kim HJ Youn CS Choi SP Wee JH Implication of cardiac marker elevation in patients who resuscitated from out-of-hospital cardiac arrest. Am J Emerg Med (2012) 30(3):464–71. 10.1016/j.ajem.2010.12.022 21296527

[9] Reichlin T Hochholzer W Stelzig C Laule K Freidank H Morgenthaler NG Incremental value of copeptin for rapid rule out of acute myocardial infarction. J Am Coll Cardiol (2009) 54(1):60–8. 10.1016/j.jacc.2009.01.076 19555842

[10] Wildi K Zellweger C Twerenbold R Jaeger C Reichlin T Haaf P Incremental value of copeptin to highly sensitive cardiac Troponin I for rapid rule-out of myocardial infarction. Int J Cardiol (2015) 190:170–6. 10.1016/j.ijcard.2015.04.133 25918073

[11] Zipes DP Wellens HJ Death SC Sudden cardiac death. Circulation (1998) 98(21):2334–51. 10.1161/01.CIR.98.21.2334 9826323

[12] Ponikowski P Voors AA Anker SD Bueno H Cleland JGF Coats AJS 2016 ESC Guidelines for the diagnosis and treatment of acute and chronic heart failure. Eur Heart J (2016) 37(27):2129–200. 10.1093/eurheartj/ehw128 27206819

[13] Reichlin T Twerenbold R Reiter M Steuer S Bassetti S Balmelli C Introduction of high-sensitivity troponin assays: impact on myocardial infarction incidence and prognosis. Am J Med (2012) 125(12):1205–13. 10.1016/j.amjmed.2012.07.015 23164485

[14] Khan SQ Dhillon OS O'Brien RJ Struck J Quinn PA Morgenthaler NG C-terminal provasopressin (copeptin) as a novel and prognostic marker in acute myocardial infarction: Leicester Acute Myocardial Infarction Peptide (LAMP) study. Circulation (2007) 115(16):2103–10. 10.1161/CIRCULATIONAHA.106.685503 17420344

[15] Narayan H Dhillon OS Quinn PA Struck J Squire IB Davies JE C-terminal provasopressin (copeptin) as a prognostic marker after acute non-ST elevation myocardial infarction: Leicester Acute Myocardial Infarction Peptide II (LAMP II) study. Clin Sci (2011) 121(2):79–89. 10.1042/CS20100564 21309746

[16] Ristagno G Latini R Plebani M Zaninotto M Vaahersalo J Masson S Copeptin levels are associated with organ dysfunction and death in the intensive care unit after out-of-hospital cardiac arrest. Crit Care (2015) 19:132 10.1186/s13054-015-0831-y 25886856 PMC4415235

[17] Myhre PL Tiainen M Pettilä V Vaahersalo J Hagve TA Kurola J NT-proBNP in patients with out-of-hospital cardiac arrest: Results from the FINNRESUSCI Study. Resuscitation (2016) 104:12–18. 10.1016/j.resuscitation.2016.04.007 27109503

[18] Sodeck GH Domanovits H Sterz F Schillinger M Losert H Havel C Can brain natriuretic peptide predict outcome after cardiac arrest? An observational study. Resuscitation (2007) 74(3):439–45. 10.1016/j.resuscitation.2007.02.001 17451863

[19] Nagao K Hayashi N Kanmatsuse K Kikuchi S Kikushima K Watanabe K B-type natriuretic peptide as a marker of resuscitation in patients with cardiac arrest outside the hospital. Circ J (2004) 68(5):477–82. 10.1253/circj.68.477 15118292

[20] Galvani M Ferrini D Ottani F Natriuretic peptides for risk stratification of patients with acute coronary syndromes. Eur J Heart Fail (2004) 6(3):327–33. 10.1016/j.ejheart.2004.01.006 14987584

[21] Lexow K Sunde K Why Norwegian 2005 guidelines differs slightly from the ERC guidelines. Resuscitation (2007) 72(3):490–2. 10.1016/j.resuscitation.2006.07.018 17161898

[22] Jacobs I Nadkarni V Bahr J Berg RA Billi JE Bossaert L Cardiac arrest and cardiopulmonary resuscitation outcome reports, update and simplificaton of the utstein templates for resuscitation registries. Circulation (2004) 110:3385–97. 15557386 10.1161/01.CIR.0000147236.85306.15

[23] Ibanex B James S Agewall S Antunes MJ Bucciarelli-Ducci C Bueno H ESC Guidelines for the management of acute myocardial infarction in patients presenting with ST-segment elevation. Eur Heart J (2017) 2018(39):119–77. 10.1093/eurheartj/ehx393 28886621

[24] Lindner TW Deakin CD Aarsetøy H Rubertsson S Heltne JK Søreide E A pilot study of angiotensin converting enzyme (ACE) genotype and return of spontaneous circulation following out-of-hospital cardiac arrest. Open Heart (2014) 1(1):e000138 10.1136/openhrt-2014-000138 25332829 PMC4189251

[25] The Norwegian Cardiac Arrest Registry. (2015). Available at: https://www.kvalitetsregistre.no/registers/norsk-hjertestansregister

[26] Lindner KH Haak T Keller A Bothner U Lurie KG Release of endogenous vasopressors during and after cardiopulmonary resuscitation. Heart (1996) 75(2):145–50. 10.1136/hrt.75.2.145 8673752 PMC484250

[27] Talwar S Squire IB Downie PF Mccullough AM Campton MC Davies JE Profile of plasma N-terminal proBNP following acute myocardial infarction; correlation with left ventricular systolic dysfunction. Eur Heart J (2000) 21(18):1514–21. 10.1053/euhj.1999.2045 10973765

[28] Sabatine MS Morrow DA de Lemos JA Omland T Desai MY Tanasijevic M Acute changes in circulating natriuretic peptide levels in relation to myocardial ischemia. J Am Coll Cardiol (2004) 44(10):1988–95. 10.1016/j.jacc.2004.07.057 15542281

[29] Nagao K Kanmatsuse K Watanabe I Ouguchi S Kikuchi S Shioiri K The diagnostic and prognostic values of a BNP in patients with out-of-hospital cardiac arrest. J Jpn Soc Internal Med (2002) 91:192.

[30] Smit B Spoelstra-de Man AM Girbes AR de Waard MC NT-proBNP in cardiopulmonary resuscitated patients treated with mild therapeutic hypothermia is not independently associated with mortality: a retrospective observational study. BMC Anesthesiol (2015) 15:48 10.1186/s12871-015-0023-y 25883532 PMC4399224

[31] Jernberg T Stridsberg M Venge P Lindahl B N-terminal pro brain natriuretic peptide on admission for early risk stratification of patients with chest pain and no ST-segment elevation. J Am Coll Cardiol (2002) 40(3):437–45. 10.1016/S0735-1097(02)01986-1 12142108

[32] Galvani M Ottani F Oltrona L Ardissino D Gensini GF Maggioni AP N-terminal pro-brain natriuretic peptide on admission has prognostic value across the whole spectrum of acute coronary syndromes. Circulation (2004) 110(2):128–34. 10.1161/01.CIR.0000134480.06723.D8 15197143

