# Supplementary material for: Initial Phase NT-proBNP, but Not Copeptin and High-Sensitivity Cardiac Troponin-T Yielded Diagnostic and Prognostic Information in Addition to Clinical Assessment of Out-of-Hospital Cardiac Arrest Patients With Documented Ventricular Fibrillation
Source: Front Cardiovasc Med. 2018 Jun 7;5:44. doi: 10.3389/fcvm.2018.00044 (PMC6001003; doi:10.3389/fcvm.2018.00044)
Supplement: Supplementary file 1 [file Table1.PDF]

Table 1. Baseline characteristics of patients suffering out-of-hospital cardiac arrest associated with documented ventricular fibrillation

	Group 1 (n=43)	Group 2 (n= 10)	Group 4 (n= 18)	p-value
Men	37 (86%)	10 (100%)	17 (94%)	0.617 [1]
Age, years	58 (49 – 66)[1]	70 (60 – 78)[1]	74 (66 – 81)[1]	<0.001 [2]
BMI, kg/m^2 (mean)	27.8	26.4[2]	28.3[3]	0.755 [3]
**Symptoms prior to SCA**				0.002 [1]
Chest pain	24 (56%)	5 (50%)	1 (6%)	
Dyspnoea	1 (2%)	0	2 (11%)	
Palpitations/syncope	0	0	0	
Asymptomatic	5 (12%)	3 (30%)	6 (33%)	
Unknown	13 (30%)	2 (20%)	9 (50%)	
**ECG findings**				0.3929 [1]
STEMI	29 (60%)	6 (60%)	0	
NSTEMI	7 (16%)	3 (30%)	0	
Ejection fraction, %	50 (40-60)[1,4]	30 (30-35)[1,2]	35 (23 – 50)[1,5]	0.024 [2]
**Coronary angiography**				0.022 [1]
Normal	0[6]	0[7]	2 (17%)[3]	
1-vessel disease	19 (53%)[6]	1 (11%)[7]	3 (25%)[3]	
2-vessel disease	8 (22%)[6]	3 (33%)[7]	1 (8%)[3]	
3-vessel disease	9 (25%)[6]	5 (56 %)[7]	6 (50%)[3]	
**Coronary intervention**				
LAD	21 (58%)[6]	5 (56%)[7]	1 (8%)[3]	0.007 [1]
CX	9 (25%)[6]	2 (22%)[7]	0[3]	0.179 [1]
RCA	6 (17%)[6]	1 (11%)[7]	1 (8%)[3]	0.865 [1]
Hypothermic treatment	27 (69%)[8]	7 (70%)	12 (67%)[9]	0.730 [1]
Implantation of ICD	1 (2%)	0	9 (50%)	<0.001 [1]
Death prior to discharge	15 (35%)	6 (60%)	9 (50 %)	0.261 [1]
**Previous history**				

Angina pectoris	5 (14%)[10]	0[7]	3 (19%)[11]	0.422 [1]
Myocardial infarction	0[13]	10 (100%)	11 (61%)	<0.001 [1]
Heart failure	0[13]	1 (11%)[8]	15 (83%)	<0.001 [1]
Previous CABG	0[13]	2 (20%)	4 (22%)	0.003 [1]
Previous PCI	1 (2%)[13]	4 (40%)	3 (17%)	0.002 [1]
Hypertension	15 (38%)[14]	4 (44%)[7]	12[15] (17%)	0.077 [1]
Mitral insufficiency	1 (3%)[8]	2 (25%)[5]	12 (67%)	<0.001 [1]
Diabetes mellitus	4 (10%)[8]	2 (22%)[7]	3 (17%)	0.540 [1]
Hypercholesterolemia	22 (54%)[16]	5 (56%)[7]	5 (31%)[11]	0.287 [1]
Smoking				0.816 [1]
Current smoking	9 (30%)	2 (29%)	4 (29%)	
Ex-smoker	17 (57%)[17]	4 (57%)[18]	6 (43%)[19]	
Family history	17 (68%)[4]	4 (57%)[18]	6 (55%)[20]	0.680 [1]
**Medication prior to admission**				
Beta-blocker	2 (6%)[21]	2 (29%)[18]	10(56%)	<0.001 [1]
Ca-blocker	6 (18%)[21]	2 (29%)[18]	6 (33%)	0.453 [1]
ACEI/ARB	4 (12%)[21]	2 (29%)[18]	15 (83%)	<0.001 [1]
Diuretics	3 (9%)[21]	0[18]	13 (72%)	<0.001 [1]
ASA	2 (6%)[21]	6 (86%)[18]	7 (39%)	<0.001 [1]
Warfarin	1 (3%)[21]	0[18]	9 (50%)	<0.001 [1]
Statins	6 (18%)[21]	5 (63%)[5]	13 (72%)	<0.001 [1]
Anti-arrhythmics	0	0[18]	0	N/A
**Baseline blood samples (median, Q1-Q3))**				
Potassium (mmol/L)	3.9[22] (3.5-4.1)	3.8[7] (3.4-4.3)	4.1[19] (3.2-4.5)	0.736 [2]
Creatinine (umol/L)	97 (83-112)	111 (102-120)	123 (93-145)	0.010 [2]
Total-cholesterol (mmol/L)	5.1 (4.1-6.6)	3.9 (3.5-5.0)	3.8 (3.2-4.2)	<0.001 [2]

Glucose (mmol/L)	14.1[2]	13.2[5]	13.5[15]	0.580 [2]
	(10.2-18.5)	(9.4-21.5)	(9.4-15.3)	
hs-CRP (mg/L)	2.1	1.7	2.7	0.122 [2]
	(1.1-4.2)	(1.1-2.0)	(1.2-12.5)	
hs-cTNT (ng/L)	116.5	40.3	52.6	0.180 [2]
	(34.6-309.6)	(17.2-201.3)	(24.45-143.5)	
Copeptin (pmol/L)	515.9[13]	879.3[5]	406.5[11]	0.034 [2]
	(243.9-918.7)	(494.7-1349)	(188.0-515.0)	
Copeptin ultrasensitive(pmol/L)	456.9	741.9	388[15]	0.054 [2]
	(204.6-761.3)	(381-1206)	(133.5-500.2)	
NT-proBNP (pmol/L)	28.2	39.1	198	0.000 [2]
	(15.7-75.6)	(10.6-113)	(102-364)	

Categorical data are given as n (%). Median values of continuous data given with 25th and 75th percentiles in parentheses (interquartile range).
ACEI, angiotensin converting enzyme inhibitor; ARB, angiotensin II receptor blocker; ASA, acetylsalisylic acid; BMI, body mass index; CABG, coronary artery bypass grafting; CAD, coronary artery disease; CX, circumflex; ECG, electrocardiography; HDL, high density lipoprotein; HF, heart failure; hsCRP, high-sensitivity C-reactive protein; ICD, implantable cardioverter defibrillator; LAD, left anterior descending artery; RCA, right coronary artery; MI, myocardial infarction; NSTEMI, non-ST-elevation myocardial; NT-proBNP, N-terminal-pro brain natriuretic peptide; PCI, percutaneous coronary intervention; SCD, sudden cardiac death; SD, standard deviation; STEMI, ST-elevation myocardial infarction; hsTnT, high-sensitivity troponin-T.
Nd = no data.
[1]median with range, [2]n=5, [3]n=12, [4]n=25, [5]n=8, [6] n=36, [7]n=9, [8]n=39, [9]n=15, [10]n=37, [11]n=16, [12]n=1, [13]n=42, [14]n=40, [15]n=17, [16]n= 41, [17] n=30, [18]n=7, [19]n=14, [20]n=11, [21]n=34, [22]n=38, [23]n=13.
[1] Fisher’s exact test, [2] Kruskal-Wallis test, [3] One-way analysis of variance
