# Supplementary figures and images for: Initial Phase NT-proBNP, but Not Copeptin and High-Sensitivity Cardiac Troponin-T Yielded Diagnostic and Prognostic Information in Addition to Clinical Assessment of Out-of-Hospital Cardiac Arrest Patients With Documented Ventricular Fibrillation
Source: Front Cardiovasc Med. 2018 Jun 7;5:44. doi: 10.3389/fcvm.2018.00044 (PMC6001003; doi:10.3389/fcvm.2018.00044)

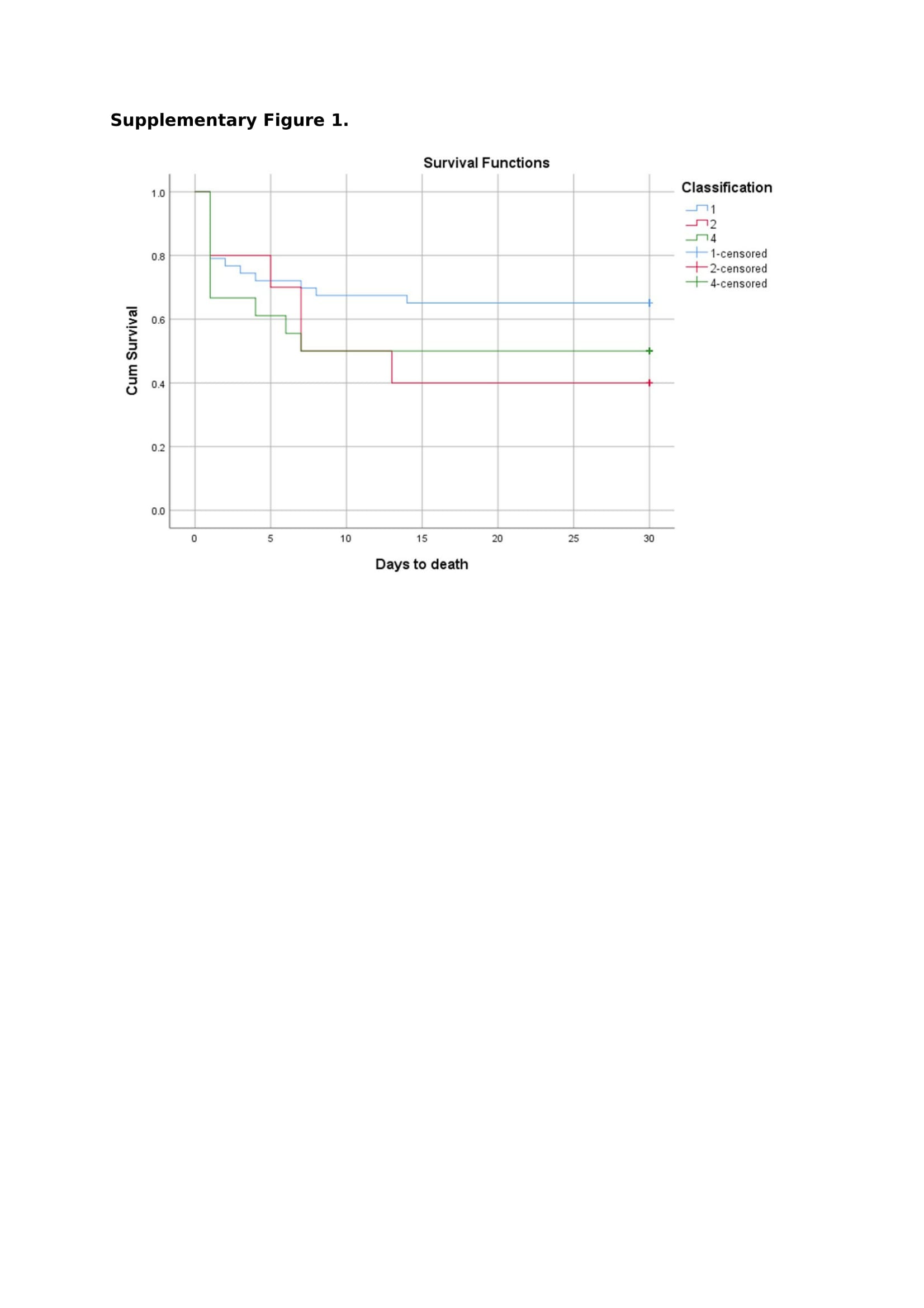

Supplement: Figure S1 — Kaplan-Meier plots for the cumulative risk for total mortality in OHCA patients, according to Group 1, 2 and 4. [file Image1.JPEG]

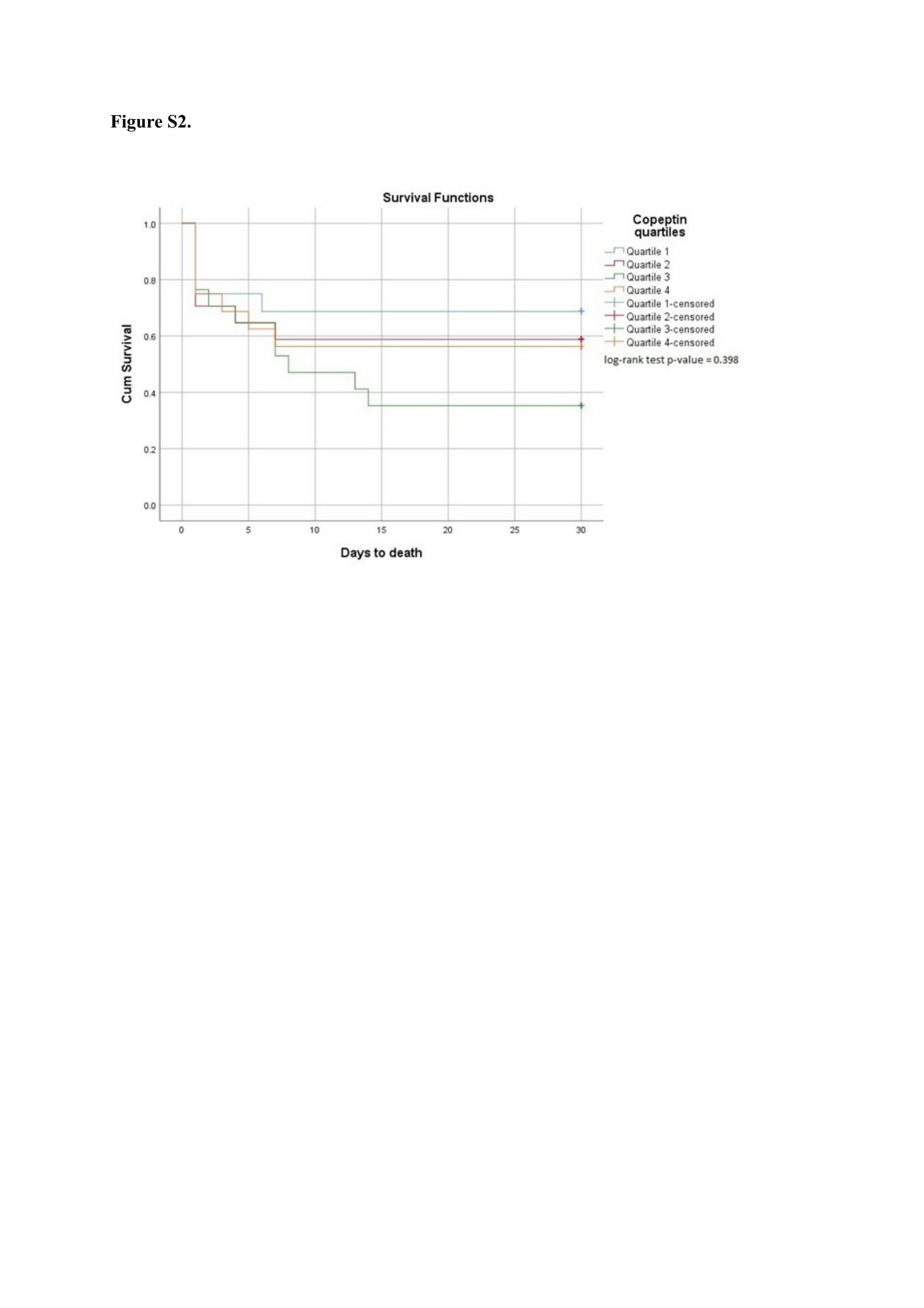

Supplement: Figure S2 — Kaplan-Meier plots for the cumulative risk for total mortality in OHCA patients according to copeptin quartiles. [file Image2.jpg]

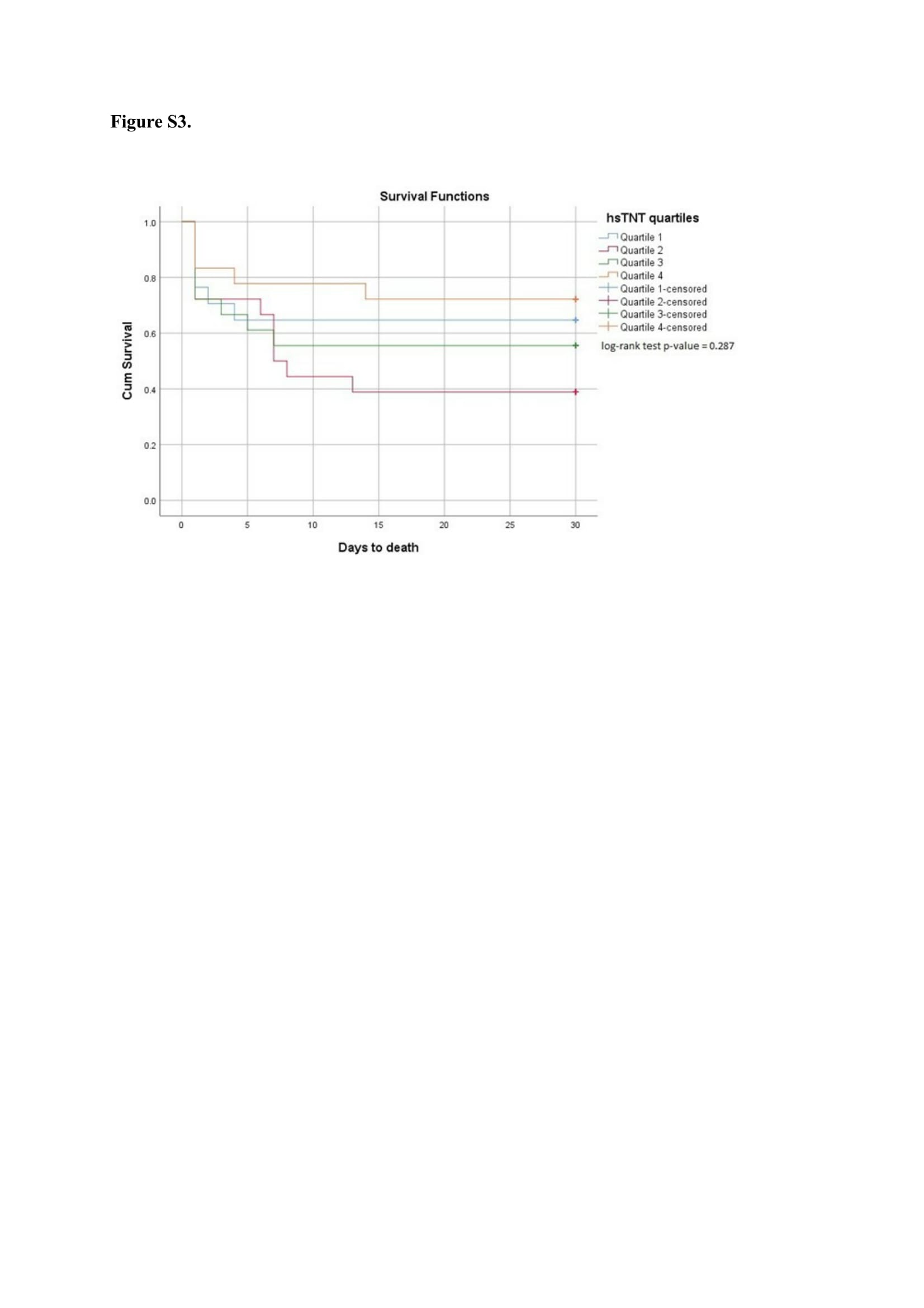

Supplement: Figure S3 — Kaplan-Meier plots for the cumulative risk for total mortality in OHCA patients according to hs-cTnT quartiles. [file Image3.jpg]

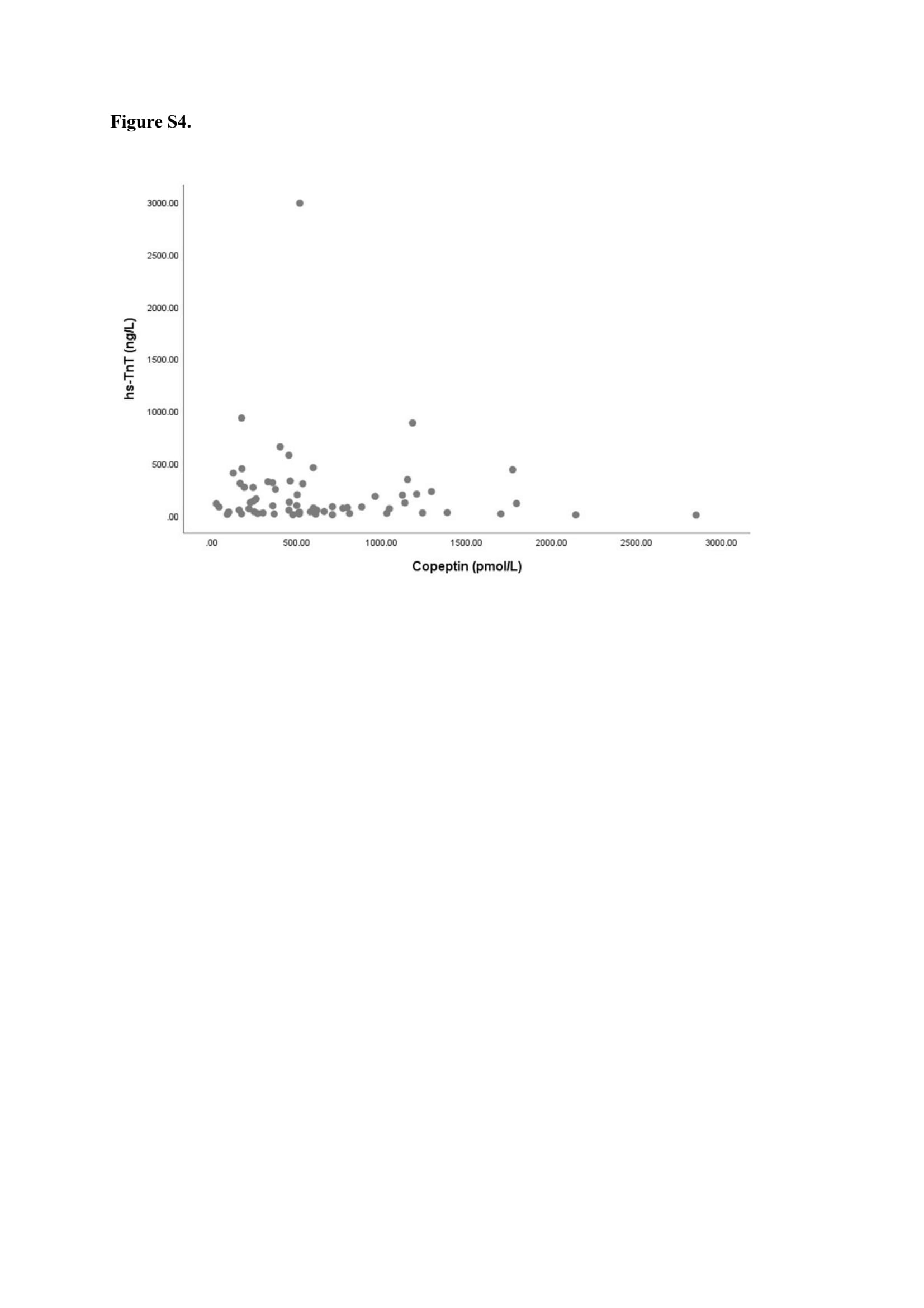

Supplement: Figure S4 — Scatter plots for hs-cTnT versus conventional copeptin. [file Image4.jpg]

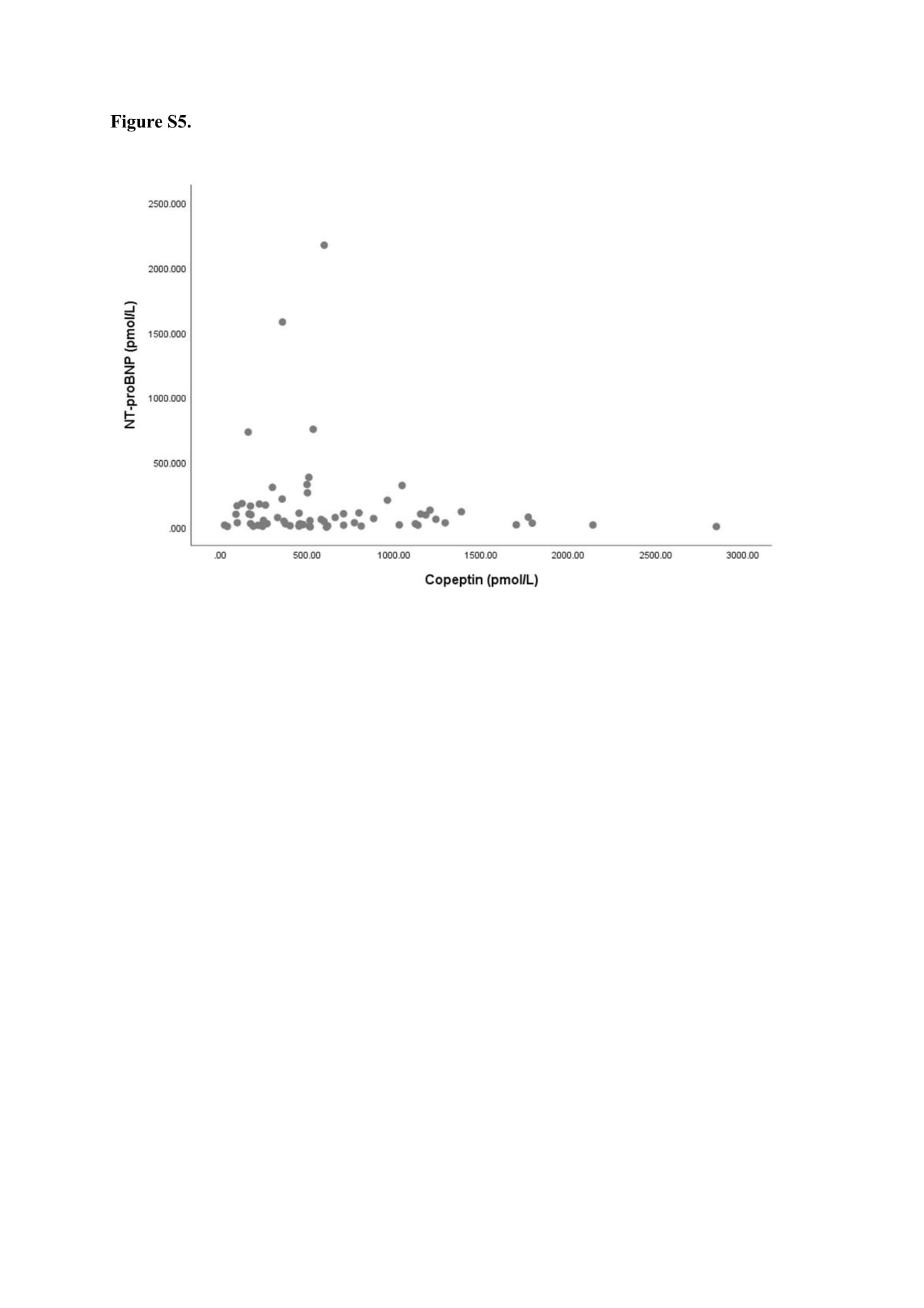

Supplement: Figure S5 — Scatter plots for NT-proBNP versus conventional copeptin. [file Image5.jpg]
